# Neutrophil count and platelet–lymphocyte ratio as simple predictors of ustekinumab response in patients with Crohn’s disease: a retrospective multicenter study

**DOI:** 10.3389/fimmu.2026.1749683

**Published:** 2026-04-22

**Authors:** Lin Li, Ping Lin, Xuemei Xu, Nianxia Qian, Shengbing Li, Mingtao Xu, Ming Deng, Xiaoming Sun, Ying Zhang, Li Xie, Ping Liu, Xiaoping Niu

**Affiliations:** 1 Department of Gastroenterology, The First Affiliated Hospital of Wannan Medical College, Wuhu, China; 2 Department of Gastroenterology, The First People’s Hospital of Wuhu, Wuhu, China; 3 Department of Gastroenterology, The First Affiliated Hospital of the University of Science and Technology of China, Hefei, China; 4 Department of Gastroenterology, Tongling Municipal Hospital, Tongling, China; 5 Department of Gastroenterology, Anqing Municipal Hospital, Anqing, China; 6 Department of Gastroenterology, The First Affiliated Hospital of Bengbu Medical University, Bengbu, China

**Keywords:** baseline neutrophil count, baseline platelet-lymphocyte ratio, clinical remission, Crohn’s disease, ustekinumab

## Abstract

**Introduction:**

Although ustekinumab (UST) has been recommended by guidelines as a parallel first-line biologic agent for Crohn’s disease (CD), its efficacy varies among individuals. This study aimed to identify baseline clinical features and laboratory indicators that can predict clinical remission with UST to assist clinical decision-making.

**Methods:**

This retrospective study collected data from patients with CD who received UST induction and maintenance therapy across multiple centers. The primary endpoint was the rate of clinical remission at week 48. Independent predictors of clinical remission were identified by multivariate logistic regression analysis. Receiver operating characteristic (ROC) curves and the Youden index were used to determine optimal cutoff values.

**Results:**

Among the 157 included patients, clinical and endoscopic remission rates were 76.4% (120/157) and 51.6% (81/157), respectively. Multivariate analysis showed that baseline neutrophil count and baseline platelet–lymphocyte ratio (PLR) were independent predictors of clinical remission at week 48 with UST treatment. Area under the ROC curve values for baseline neutrophil count, baseline PLR, and their combination were 0.633, 0.709, and 0.769, respectively. Optimal cutoff values for baseline neutrophil count and baseline PLR were ≤4.135×10^9^/L and <237.912, respectively.

**Discussion:**

Baseline neutrophil count and baseline PLR together constitute a simple and effective predictive model. This model may help identify patients who are more responsive to UST before treatment, offering a practical tool for individualized and precise treatment of CD.

## Introduction

Crohn’s disease (CD) is a chronic, relapsing, and disabling type of inflammatory bowel disease with a complex pathogenesis that involves immune dysregulation (1). Therapeutic goals for CD have substantially evolved over the past decade, shifting from symptom control toward long-term endpoints such as corticosteroid-free clinical remission, endoscopic healing, and prevention of disease progression. The emergence of biologic agents has greatly increased the likelihood of achieving these goals. Among biologics, anti-tumor necrosis factor-alpha (anti-TNF-α) agents, such as infliximab and adalimumab, have long been the cornerstone of treatment for moderate-to-severe CD (2–4). Ustekinumab (UST), a monoclonal antibody targeting the p40 subunit of interleukin (IL)-12 and IL-23, has demonstrated robust efficacy and a favorable safety profile in clinical trials and real-world settings. It is now an established option for patients with moderate-to-severe disease, including those with TNF-α antagonist therapy failure (5, 6).

Current international guidelines, including those from the European Crohn’s and Colitis Organisation and the American Gastroenterological Association, now parallelly recommend UST alongside anti-TNF-α agents as a first-line biologic therapy for CD (7). Despite its overall efficacy, a substantial proportion of patients—up to 30%–40% in some cohorts—exhibit primary nonresponse to UST (8). Furthermore, the drug’s mechanism of action, which targets the IL-12/T helper (Th) 1 and IL-23/Th17 pathways, indicates that it may be particularly effective in specific patient endotypes that have not been clearly defined (9). The inability to identify these “optimal responders” *a priori* leads to a trial-and-error approach, potentially exposing patients to periods of ineffective treatment, delayed disease control, and unnecessary healthcare costs.

Consequently, there is an urgent unmet need for reliable and readily accessible biomarkers that can predict response to UST prior to treatment initiation. Current strategies, such as therapeutic drug monitoring (10), are valuable for optimizing therapy after initiation; they remain reactive, rather than predictive. Investigations into clinical predictors have yielded inconsistent results. Whereas some studies suggest that prior anti-TNF-α failure is associated with a diminished response to UST, others indicate that UST remains effective in this population (11, 12). Similarly, disease behavior (e.g., stricturing or penetrating) and location may influence outcomes, but evidence is not sufficiently conclusive to guide individual patient decisions (13). Complex scoring systems or multi-omics signatures, although promising, often lack the simplicity and immediacy required for routine clinical implementation.

In this context, routine laboratory parameters offer a compelling alternative. As inexpensive, widely available, and objective measures of systemic inflammation and immune status, they hold promise for pragmatic clinical prediction. Accordingly, this study aimed to determine pretreatment predictors of UST response by systematically evaluating clinical response and remission rates in patients with CD during induction and maintenance phases across multiple centers, and to pre-identify these “best responders”.

## Materials and methods

### Study design and patient population

This multicenter, retrospective, observational cohort study was conducted across six tertiary medical centers: the First Affiliated Hospital of Wannan Medical College, the First People’s Hospital of Wuhu, the First Affiliated Hospital of the University of Science and Technology of China, the First Affiliated Hospital of Bengbu Medical University, Anqing Municipal Hospital, and Tongling Municipal Hospital. The study protocol was conducted in accordance with the Declaration of Helsinki and was approved by the Ethics Committee of the First Affiliated Hospital of Wannan Medical College (No. 2024SR219).

We systematically screened the electronic medical records of patients with a confirmed diagnosis of CD who initiated UST treatment between January 2017 and December 2025. Inclusion criteria were as follows (1); age 18–75 years (2); diagnosis of CD based on the 2018 Beijing consensus on the diagnosis and treatment of inflammatory bowel disease (14) (3); active disease at baseline, defined as a Crohn’s Disease Activity Index (CDAI) score >150 (4); completion of the standard UST induction regimen, consisting of a single intravenous weight-based dose (~6 mg/kg) at week 0, followed by a subcutaneous 90 mg dose at week 8, with a minimum follow-up duration of 48 weeks after the first UST dose; and (5); availability of complete baseline laboratory data, including data at the first infusion of UST (week 0) and laboratory and auxiliary examination data within the subsequent 48 weeks. Exclusion criteria were as follows (1); concurrent active infection (e.g., *Clostridioides difficile*, cytomegalovirus) or other conditions that could substantially confound inflammatory markers or disease assessment (e.g., concurrent malignancy, major surgery within the previous 8 weeks) (2); more than 30% missing data or loss to follow-up before the week 48 assessment; and (3); concurrent use of other biologic agents with UST.

### Data collection

Data were extracted from the electronic medical records of each center using a standardized, pre-piloted case report form. Demographic data, disease characteristics (including disease duration, CDAI, Simple Endoscopic Score for Crohn’s Disease [SES-CD], behavior according to the Montreal classification, and prior medical and surgical history), and baseline laboratory data were collected. Laboratory indicators were recorded at weeks 0, 8, 16, 24, and 48 after UST initiation, including platelet count, hemoglobin, neutrophil count, eosinophil count, lymphocyte count, monocyte count, albumin, C-reactive protein (CRP), erythrocyte sedimentation rate, fecal calprotectin, platelet–lymphocyte ratio (PLR), monocyte–lymphocyte ratio, neutrophil–lymphocyte ratio (NLR), and systemic immune-inflammation index (SII).

### Outcomes and definitions

The primary outcome was clinical remission at week 48, defined as a CDAI score ≤ 150. Key secondary outcomes were endoscopic remission at week 48 and clinical response at week 8. Endoscopic remission at week 48 was defined as SES-CD ≤3 for patients with a baseline SES-CD above 3, or SES-CD = 0 for patients with a baseline SES-CD of 3. Clinical response at week 8 was defined as CDAI ≤150 or a CDAI-100 response (≥100-point decrease in CDAI score) (15).

Other secondary outcomes were glucocorticoid-free response at week 48 (CDAI score <150 without glucocorticoid use for at least 30 days) and clinical remission rates at weeks 16 and 24. All adverse events were recorded.

**Figure 1:**
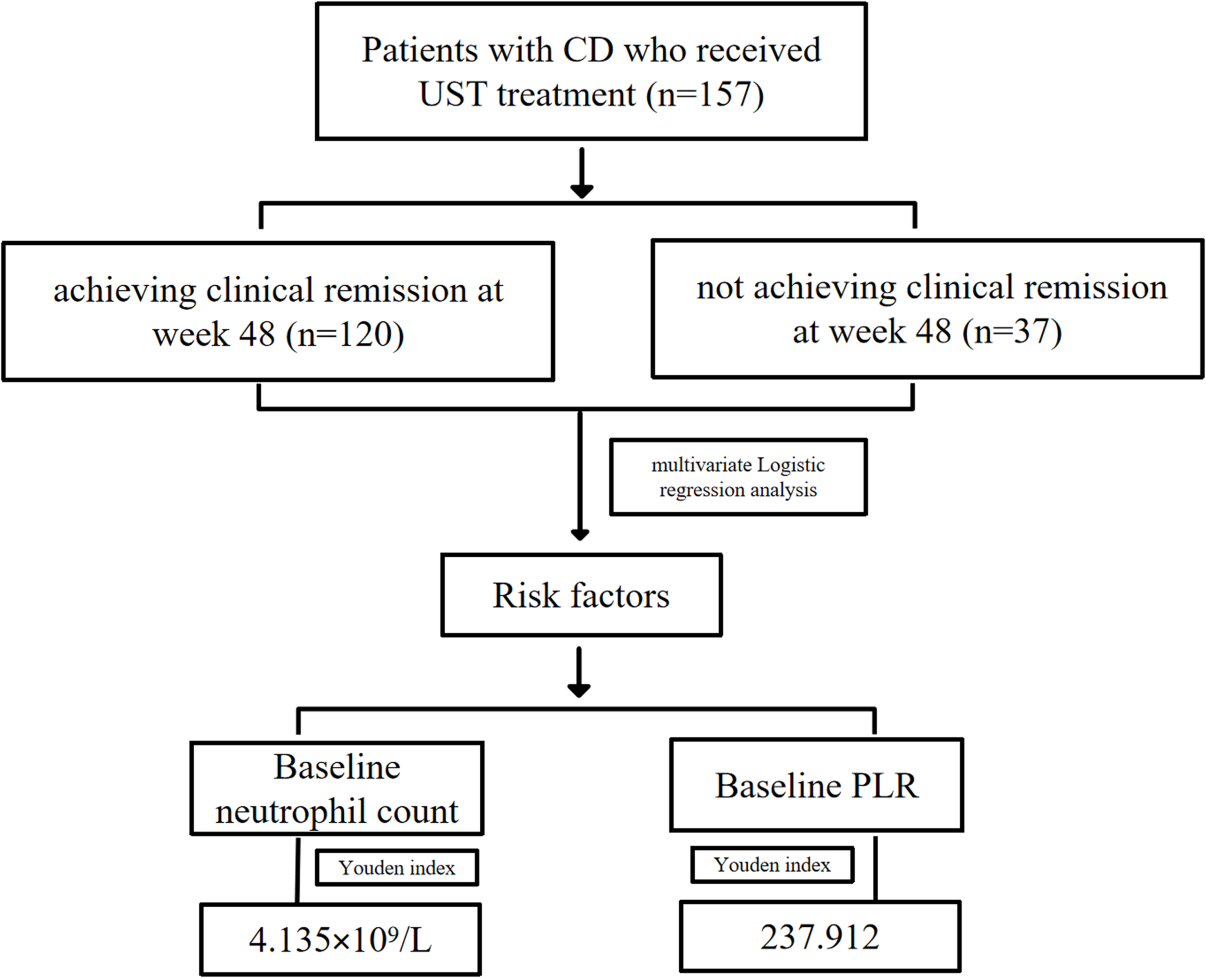
Flow chart of this study. CD, Crohn’s disease; UST, ustekinumab; PLR, platelet–lymphocyte ratio.

Patients were categorized as “responders” (achieving clinical remission at week 48) and “non-responders” (not achieving remission) for the primary analysis (**Figure 1**). Subgroup analyses were performed based on baseline neutrophil count and baseline PLR in patients with previous anti-TNF-α treatment failure and those who initially received UST treatment. For secondary analysis, patients were categorized as “rapid responders” (achieving clinical response at week 8) and “non-rapid responders” (not achieving clinical response).

### Statistical analysis

Continuous variables were presented as mean ± standard deviation or median (interquartile range) and compared using Student’s t-test or the Mann–Whitney U test, as appropriate. Categorical variables were expressed as frequencies (%) and compared using the chi-square test or Fisher’s exact test. Multivariate logistic regression analysis was used to identify independent predictors of clinical remission at week 48. The predictive value of independent risk factors was evaluated using receiver operating characteristic (ROC) curves, and the Youden index was used to determine optimal cutoff values. Two-sided *p*-values of <0.05 were considered statistically significant. All analyses were performed using SPSS 26.0 (SPSS Inc., Chicago, IL, USA) or GraphPad Prism 8.0 (GraphPad Software, San Diego, CA, USA).

## Results

### Baseline population characteristics

In total, 157 patients with CD who received UST therapy and met the inclusion criteria were included in the final analysis. Of these, 99 (63.1%) were men and 58 (36.9%) were women. Disease duration ranged from 0.5 to 23 years, with a mean of 4.9 ± 4.1 years.

According to the Montreal classification, most patients were classified as A2 (17–40 years old) (111 cases, 70.7%), L3 (ileocolonic type) (118 cases, 75.2%), and B1 (non-stricturing and non-penetrating type) (84 cases, 53.5%). Among these, 82 patients (52.2%) displayed perianal disease. Enteral nutrition was used in 70 cases (44.6%), 62 patients (39.5%) had a history of ileocolonic surgery, and six patients (3.8%) showed extraintestinal manifestations. UST was used as first-line therapy in 55 cases (35%) and as second-line therapy in 102 cases (65%). Baseline laboratory data and clinical characteristics are summarized in **Table 1**.

**Table 1:** Basic information of the enrolled patients.

Variables	n (%)
Gender	
Male	99 (63.1)
Female	58 (36.9)
Age at onset	
A1	7 (4.5)
A2	111 (70.7)
A3	39 (24.8)
Location	
L1	29 (18.5)
L2	7 (4.5)
L3	118 (75.2)
L4	3 (1.9)
Disease behavior	
B1	84 (53.5)
B2	51 (32.5)
B3	22 (14)
P	82 (52.2)
Disease duration (years)	4.9 ± 4.1
Combined enteral nutrition	
Present	70 (44.6)
Absent	87 (55.4)
Prior surgical history	
Present	62 (39.5)
Absent	95 (60.5)
Extraintestinal manifestations	
Present	6 (3.8)
Absent	151 (96.2)
UST used as first-line therapy	
Yes	55 (35.0)
No	102 (65.0)
Baseline BMI (kg/m^2^)	19.6 ± 2.7
Baseline ALB (g/L)	39.6 ± 5.8
PLT (×10^9^/L)	280.6 ± 97.3
Baseline Hb (g/L)	123.6 ± 22.1
Baseline lymphocyte count (×10^9^/L)	1.4 ± 0.6
Baseline monocyte count (×10^9^/L)	0.4 [0.3, 0.6]
Baseline neutrophil count (×10^9^/L)	3.8 ± 1.6
Baseline eosinophil count (×10^9^/L)	0.09 [0.06, 0.18]
Baseline CDAI (scores)	210.2 ± 51.8
Baseline CRP (mg/L)	8.0 [4.5, 21.4]
Baseline ESR (mm/h)	18.0 [10.0, 27.5]
Baseline calprotectin (μg/g)	210.0 [151.5, 261.5]
Baseline PLR	236.6 ± 202.3
Baseline MLR	0.3 [0.2, 0.4]
Baseline NLR	3.1 ± 1.9
Baseline SII	715.4 (485.1, 1033.3)
Baseline SES-CD (scores)	9.1 ± 5.4

UST, Ustekinumab; BMI, body mass index; ALB, albumin; PLT, platelet; Hb, hemoglobin; CDAI, Crohn’s Disease Activity Index; CRP, C-reactive protein; ESR, erythrocyte sedimentation rate; PLR, platelet–lymphocyte ratio; MLR, monocyte–lymphocyte ratio; NLR, neutrophil–lymphocyte ratio; SII, systemic immune-inflammation index; SES-CD, Simple Endoscopic Score for Crohn’s Disease.

### Univariate and multivariate logistic regression analysis of risk factors for clinical remission at week 48 with UST treatment

At week 48, 120 patients achieved clinical remission after UST treatment, corresponding to a remission rate of 76.4% (120/157). Endoscopic remission was achieved in 81 of 157 patients (51.6%).

As shown in **Table 2**, univariate analysis indicated that baseline platelet count, baseline neutrophil count, baseline erythrocyte sedimentation rate, baseline PLR, baseline NLR, and baseline SII were significantly associated with clinical remission at week 48 (*p* < 0.05). By contrast, sex, age at onset, lesion location, disease behavior, disease duration, enteral nutrition, prior surgical history, extraintestinal manifestations, first-line use of UST, baseline body mass index, baseline albumin, baseline hemoglobin, baseline lymphocyte count, baseline monocyte count, baseline eosinophil count, baseline CDAI, baseline CRP, baseline fecal calprotectin, baseline monocyte–lymphocyte ratio, and baseline SES-CD were not significantly associated with clinical remission (*p*>0.05).

**Table 2:** Univariate analysis of risk factors for clinical remission at week 48 with UST treatment.

Variables	n	Responders	Non-responders	χ^2^	*p*
Gender				0.269	0.604
Male	99	77	22		
Female	58	43	15		
Age at onset				1.950	0.187
A1	7	5	2		
A2	111	82	29		
A3	39	33	6		
Location				2.020	0.195
L1	29	25	4		
L2	7	5	2		
L3	118	88	30		
L4	3	2	1		
Disease behavior				3.057	0.088
B1	84	68	16		
B2	51	38	13		
B3	22	14	8		
P	82	59	23		
Disease duration (years)				0.398	0.529
≦3	75	59	16		
>3	82	61	21		
Combined enteral nutrition				0.897	0.344
Present	70	51	19		
Absent	87	69	18		
Prior surgical history				0.285	0.594
Present	62	46	16		
Absent	95	74	21		
Extraintestinal manifestations				0.165	0.687
Present	6	5	1		
Absent	151	115	36		
UST used as first-line therapy				2.439	0.122
Yes	55	46	9		
No	102	74	28		
Baseline BMI (kg/m^2^)				0.077	0.782
<18.5	71	55	16		
≥6.52	86	65	21		
Baseline ALB (g/L)				3.849	0.053
<40	84	59	25		
≧40	73	61	12		
Baseline PLT (×10^9^/L)				0.396	**<0.001**
<300	101	87	14		
≧300	56	33	23		
Baseline Hb (g/L)				0.486	0.486
<130 (male) or <120 (female)	77	57	20		
≧130 (male) or ≧120 (female)	80	63	17		
Baseline lymphocyte count (×10^9^/L)				3.265	0.071
<1.1	49	33	16		
≧1.1	108	87	21		
Baseline monocyte count (×10^9^/L)				0.195	0.659
<0.4	60	47	13		
≧0.4	97	73	24		
Baseline neutrophil count (×10^9^/L)				8.046	**0.005**
<4.0	91	77	14		
≧4.0	66	43	23		
Baseline eosinophil count (×10^9^/L)				0.021	0.885
<0.2	126	96	30		
≧0.2	31	24	7		
Baseline CDAI (scores)				1.963	0.161
<220	108	86	22		
≧220	49	34	15		
Baseline CRP (mg/L)				2.216	0.330
<10	87	70	17		
≧10	69	49	20		
Baseline ESR (mm/h)				8.861	**0.003**
≦15 (male) or ≦20 (female)	76	66	10		
>15 (male) or >20 (female)	81	54	27		
Baseline calprotectin (μg/g)				2.356	0.308
<200	75	61	14		
≧200	81	58	23		
Baseline PLR				7.604	**0.006**
<200	82	70	12		
≧200	75	50	25		
Baseline MLR				0.337	0.584
<0.6	136	105	31		
≧0.6	21	15	6		
Baseline NLR				3.908	**0.048**
<3.0	94	77	17		
≧3.0	63	43	20		
Baseline SII				13.004	**<0.001**
<900	106	90	16		
≧900	51	30	21		
Baseline SES-CD				1.880	0.391
<9	65	59	14		
≧9	78	60	23		

UST, Ustekinumab; BMI, body mass index; ALB, albumin; PLT, platelet; Hb, hemoglobin; CDAI, Crohn’s Disease Activity Index; CRP, C-reactive protein; ESR, erythrocyte sedimentation rate; PLR, platelet–lymphocyte ratio; MLR, monocyte–lymphocyte ratio; NLR, neutrophil–lymphocyte ratio; SII, systemic immune-inflammation index; SES-CD, Simple Endoscopic Score for Crohn’s Disease. Bold values indicate statistical significance (p < 0.05).

A multivariate logistic regression model was then constructed using all variables. As shown in **Table 3**, baseline neutrophil count (odds ratio [OR]=0.127, 95% confidence interval [CI]: 0.026–0.608, *p* = 0.010) and baseline PLR (OR = 0.146, 95% CI: 0.033–0.655, *p* = 0.012) were identified as independent predictors of clinical remission at week 48 with UST treatment.

**Table 3:** Multivariate Logistic regression analysis of independent risk factors for clinical remission at week 48 with UST treatment.

Risk factors	B	S.E.	Wald	*p*	OR (95%CI)
Gender	0.613	0.580	1.119	0.290	1.847 (0.593-5.756)
Age at onset	0.844	1.508	0.534	0.766	2.326 (0.121-44.679)
Location	-1.213	2.143	4.301	0.231	0.297 (0.004-19.837)
Disease behavior	-1.675	0.948	3.667	0.160	0.187 (0.029-1.201)
Disease duration	0.010	0.565	0.000	0.986	1.010 (0.334-3.053)
Combined enteral nutrition	-0.959	0.692	1.921	0.166	0.383 (0.099-1.488)
Prior surgical history	0.307	0.680	0.204	0.651	1.360 (0.359-5.156)
Extraintestinal manifestations	-0.710	1.561	0.207	0.649	0.492 (0.023-10.478)
UST used as first-line therapy	-0.566	0.624	0.822	0.365	0.568 (0.167-1.931)
Baseline BMI	0.403	0.617	0.427	0.514	1.496 (0.447-5.012)
Baseline ALB	-0.574	0.685	0.702	0.402	0.563 (0.147-2.157)
Baseline PLT	-1.087	0.635	2.935	0.087	0.337 (0.097-1.170)
Baseline Hb	0.469	0.622	0.568	0.451	1.598 (0.472-5.408)
Baseline lymphocyte count	-1.050	0.736	2.037	0.154	0.350 (0.083-1.480)
Baseline monocyte count	0.153	0.652	0.055	0.815	1.165 (0.325-4.182)
Baseline neutrophil count	-2.067	0.800	6.667	**0.010**	0.127 (0.026-0.608)
Baseline eosinophil count	-0.436	0.686	0.404	0.525	0.647 (0.169-2.479)
Baseline CDAI	-0.147	0.632	0.054	0.817	1.158 (0.336-3.995)
Baseline CRP	0.897	0.643	1.951	0.163	2.453 (0.696-8.644)
Baseline ESR	-1.198	0.615	3.801	0.051	0.302 (0.090-1.006)
Baseline calprotectin	-0.124	0.189	0.427	0.513	0.884 (0.610-1.280)
Baseline PLR	-1.921	0.764	6.317	**0.012**	0.146 (0.033-0.655)
Baseline NLR	0.324	0.816	0.158	0.691	1.383 (0.279-6.845)
Baseline MLR	-1.224	0.947	1.670	0.196	0.294 (0.046-1.882)
Baseline SII	-0.359	0.945	0.144	0.705	0.699 (0.110-4.457)
Baseline SES-CD	-0.278	0.580	0.230	0.631	0.757 (0.243-2.360)

UST, Ustekinumab; BMI, body mass index; ALB, albumin; PLT, platelet; Hb, hemoglobin; CDAI, Crohn’s Disease Activity Index; CRP, C-reactive protein; ESR, erythrocyte sedimentation rate; PLR, platelet–lymphocyte ratio; MLR, monocyte–lymphocyte ratio; NLR, neutrophil–lymphocyte ratio; SII, systemic immune-inflammation index; SES-CD, Simple Endoscopic Score for Crohn’s Disease. Bold values indicate statistical significance (p < 0.05).

### Predictive value of independent risk factors for clinical remission at week 48 with UST treatment

**Figure 2:**
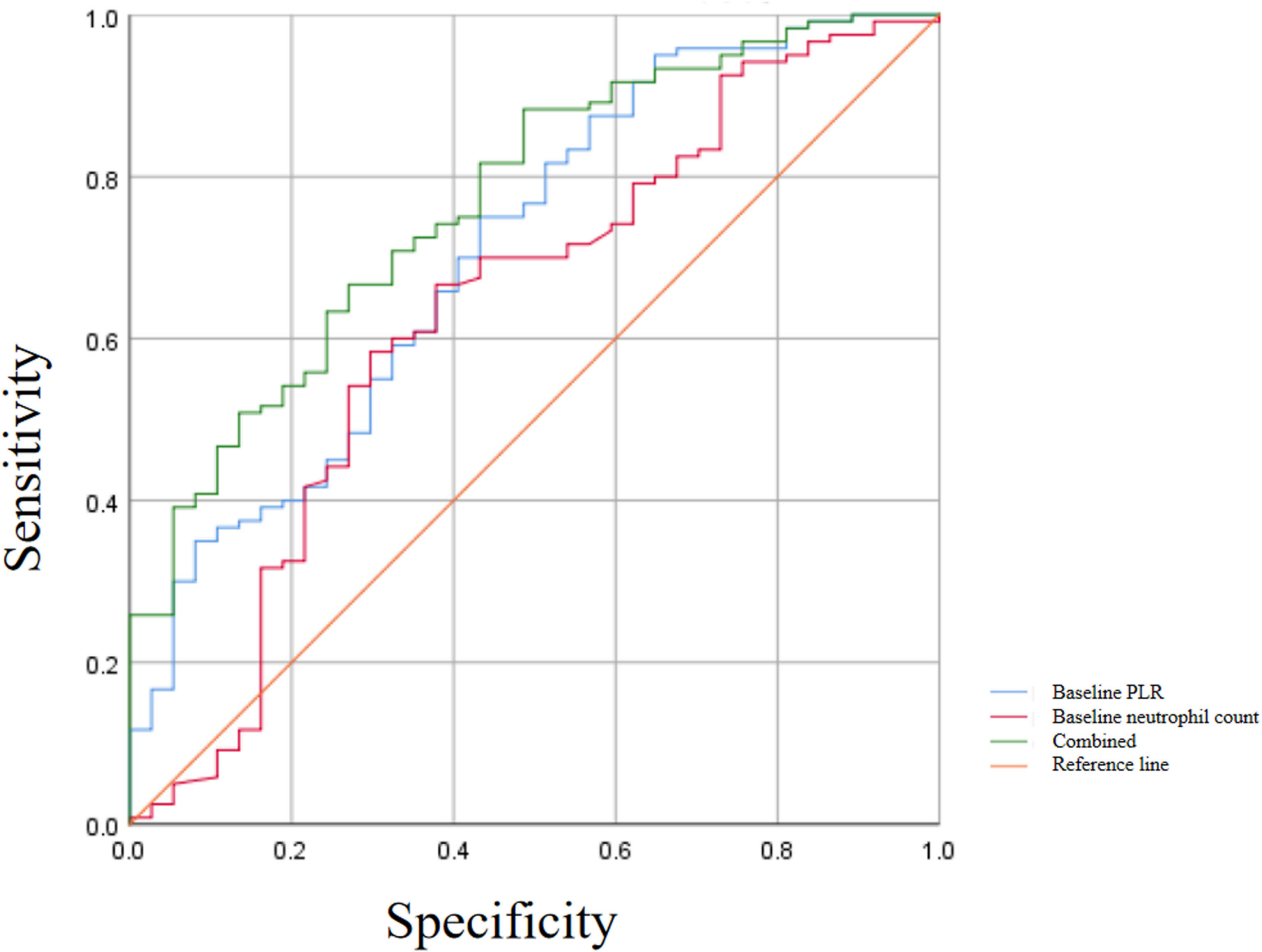
Predictive value of independent risk factors for clinical remission at week 48 with UST treatment. A combined model incorporating baseline neutrophil count and baseline PLR for detecting clinical remission at week 48 with UST treatment was 0.769 (95% CI: 0.686–0.852, *p* < 0.001), which was higher than the performance of either parameter alone. The sensitivity and specificity of the combined model were 88.3% and 51.4%, respectively.

ROC curves were used to evaluate the predictive value of independent risk factors for clinical remission at week 48 with UST treatment. As shown in **Figure 2**; **Table 4**, area under the ROC curve (AUC) values were calculated for baseline neutrophil count, baseline PLR, and their combined measurement. The AUC for baseline neutrophil count was 0.633 (95% CI: 0.523–0.742, *p* = 0.015), indicating fair to good predictive ability. The Youden index was maximized at a baseline neutrophil count of 4.135×10^9^/L, which was identified as the optimal cutoff value. At this threshold, the sensitivity was 66.7% and specificity was 62.2% for predicting clinical remission.

**Table 4:** Predictive values under the ROC curve for each independent risk factor.

Risk factors	AUC	95%CI	*P*-value	Sensitivity(%)	Specificity (%)	Youden index
Baseline neutrophil count	0.633	0.523-0.742	0.015	66.7	62.2	0.289
Baseline PLR	0.709	0.611-0.806	<0.001	75	56.8	0.318
Combined factors	0.769	0.686-0.852	<0.001	88.3	51.4	0.397

ROC; receiver operating characteristic; PLR, platelet–lymphocyte ratio; AUC, area under ROC curve.

Similarly, baseline PLR demonstrated significant predictive value, with an AUC of 0.709 (95% CI: 0.611–0.806, *p* < 0.001). The optimal cutoff value for PLR, determined by the maximum Youden index, was 237.912. This cutoff point yielded a sensitivity of 75% and a specificity of 56.8%.

The combined measurement of baseline neutrophil count and baseline PLR showed improved predictive performance, with an AUC of 0.769 (95% CI: 0.686–0.852, *p* < 0.001), which was higher than the performance of either parameter alone. The sensitivity and specificity of the combined model were 88.3% and 51.4%, respectively.

### Subgroup analysis of baseline neutrophil count and PLR in patients with previous anti-TNF-α treatment failure and patients with initial UST treatment

Based on the above results, baseline neutrophil count ≤4.135×10^9^/L and baseline PLR <237.912 may serve as reliable indicators for predicting “dominant responders” to UST. To adjust for the general observation that patients with milder inflammation tend to respond better to treatment, patients were divided into two subgroups: those with previous anti-TNF-α treatment failure and those receiving UST as initial therapy. The results showed that PLR <237.912 strongly predicted UST response in both subgroups (previous anti-TNF-α treatment failure group: OR = 0.350, 95% CI: 0.142–0.860, *p* = 0.022; initial UST treatment group: OR = 0.118, 95% CI: 0.025–0.566, *p* = 0.008), indicating consistent predictive value across subgroups. By contrast, baseline neutrophil count ≤4.135×10^9^/L was significant only in the previous anti-TNF-α treatment failure group (OR = 0.228, 95% CI: 0.091–0.572, *p* = 0.002) and not in the initial UST treatment group (OR = 1.687, 95% CI: 0.401–7.096, *p* = 0.475). Such findings suggest that, rather than predicting a universally favorable response, these markers can identify patients who remain sensitive to UST after anti-TNF-α treatment failure.

### Univariate and multivariate logistic regression analysis of risk factors for rapid clinical response at week 8 with UST treatment

Univariate and multivariate regression analyses (including all variables) were performed to identify predictors of rapid clinical response at week 8 with UST treatment. As shown in **Table 5**, univariate analysis indicated that age at onset, baseline PLR, and baseline SII were significantly associated with rapid clinical response (*p* < 0.05). Multivariate analysis demonstrated that age at onset (OR = 4.010, 95% CI: 1.116–14.412, *p* = 0.027) and baseline CDAI (OR = 5.137, 95% CI: 1.378–19.157, *p* = 0.015) were independent predictors of rapid clinical response at week 8 (**Table 6**).

**Table 5:** Univariate analysis of risk factors for clinical response at week 8 with UST treatment.

Variables	n	Rapid responders	Non-rapid responders	χ^2^	*P*
Gender				1.355	0.244
Male	99	25	74		
Female	58	10	48		
Age at onset				6.169	**0.046**
A1	7	3	4		
A2	111	19	92		
A3	39	13	26		
Location				2.046	0.563
L1	29	6	23		
L2	7	3	4		
L3	118	25	93		
L4	3	1	2		
Disease behavior				1.296	0.523
B1	84	16	68		
B2	51	14	37		
B3	22	5	17		
P	82	18	64		
Disease duration (years)				1.090	0.296
≦3	75	14	61		
>3	82	21	61		
Combined enteral nutrition				1.010	0.315
Present	70	13	57		
Absent	87	22	65		
Prior surgical history				0.511	0.475
Present	62	12	50		
Absent	95	23	72		
Extraintestinal manifestations				0.114	1.000
Present	6	1	5		
Absent	151	34	117		
UST used as first-line therapy				0.088	0.766
Yes	55	13	42		
No	102	22	80		
Baseline BMI (kg/m^2^)				0.496	0.481
<18.5	71	14	57		
≥7.51	86	21	65		
Baseline ALB (g/L)				2.052	0.152
<40	84	15	69		
≧40	73	20	53		
Baseline PLT (×10^9^/L)				1.945	0.163
<300	101	26	75		
≧300	56	9	47		
Baseline Hb (g/L)				0.495	0.566
<130 (male) or <120 (female)	77	19	58		
≧130 (male) or ≧120 (female)	80	16	64		
Baseline lymphocyte count (×10^9^/L)				1.464	0.226
<1.1	49	8	41		
≧1.1	108	27	81		
Baseline monocyte count (×10^9^/L)				0.061	0.805
<0.4	60	14	46		
≧0.4	97	21	76		
Baseline neutrophil count (×10^9^/L)				1.111	0.292
<4.0	91	23	68		
≧4.0	66	12	54		
Baseline eosinophil count (×10^9^/L)				0.192	0.661
<0.2	126	29	97		
≧0.2	31	6	25		
Baseline CDAI (scores)				4.152	0.061
<220	108	29	79		
≧220	49	6	43		
Baseline CRP (mg/L)				0.618	0.734
<10	87	21	66		
≧10	69	14	55		
Baseline ESR (mm/h)				0.165	0.685
≦15 (male) or ≦20 (female)	76	18	58		
>15 (male) or >20 (female)	81	17	64		
Baseline calprotectin (μg/g)				3.777	0.151
<200	75	15	60		
≧200	81	19	62		
Baseline PLR				4.821	**0.028**
<200	82	24	58		
≧200	75	11	64		
Baseline MLR				0.147	1.000
<0.6	136	31	105		
≧0.6	21	4	17		
Baseline NLR				2.504	0.114
<3.0	94	25	69		
≧3.0	63	10	53		
Baseline SII				4.833	**0.028**
<900	106	29	77		
≧900	51	6	45		
Baseline SES-CD				0.571	0.752
<9	65	58	7		
≧9	78	63	15		

UST, Ustekinumab; BMI, body mass index; ALB, albumin; PLT, platelet; Hb, hemoglobin; CDAI, Crohn’s Disease Activity Index; CRP, C-reactive protein; ESR, erythrocyte sedimentation rate; PLR, platelet–lymphocyte ratio; MLR, monocyte–lymphocyte ratio; NLR, neutrophil–lymphocyte ratio; SII, systemic immune-inflammation index; SES-CD, Simple Endoscopic Score for Crohn’s Disease. Bold values indicate statistical significance (p < 0.05).

**Table 6:** Multivariate Logistic regression of risk factors for clinical response at week 8 with UST treatment.

Risk factors	B	S.E.	Wald	*p*	OR (95%CI)
Gender	1.086	0.617	3.103	0.078	2.963 (0.885-9.919)
Age at onset	1.389	0.653	7.190	**0.027**	4.010 (1.116-14.412)
Location	1.345	1.913	1.840	0.606	3.838 (0.090-163.061)
Disease behavior	0.163	0.566	1.312	0.519	1.177 (0.396-3.503)
Disease duration	0.525	0.527	0.991	0.320	1.690 (0.601-4.752)
Combined enteral nutrition	0.412	0.595	0.478	0.489	1.510 (0.470-4.850)
Prior surgical history	0.899	0.605	2.204	0.138	2.456 (0.750-8.044)
Extraintestinal manifestations	0.905	1.485	0.371	0.542	2.472 (0.135-45.379)
UST used as first-line therapy	0.232	0.594	0.153	0.696	1.262 (0.394-4.038)
Baseline BMI	-0.660	0.565	1.367	0.242	0.517 (0.171-1.563)
Baseline ALB	0.071	0.598	0.014	0.905	1.074 (0.333-3.468)
Baseline PLT	-0.332	0.643	0.266	0.606	0.718 (0.203-2.530)
Baseline Hb	1.049	0.588	3.182	0.074	2.855 (0.902-9.039)
Baseline lymphocyte count	-0.323	0.725	0.198	0.656	0.724 (0.175-2.988)
Baseline monocyte count	-0.547	0.636	0.740	0.390	0.579 (0.166-2.012)
Baseline neutrophil count	-0.930	0.697	1.778	0.182	0.395 (0.101-1.548)
Baseline eosinophil count	0.536	0.680	0.622	0.430	1.709 (0.451-6.480)
Baseline CDAI	1.637	0.672	5.939	**0.015**	5.137 (1.378-19.157)
Baseline CRP	-0.147	0.681	0.046	0.830	0.864 (0.227-3.281)
Baseline ESR	0.479	0.609	0.618	0.432	1.614 (0.489-5.323)
Baseline calprotectin	-0.255	0.240	1.129	0.288	0.775 (0.485-1.240)
Baseline PLR	-1.361	0.713	3.643	0.056	0.256 (0.063-1.037)
Baseline NLR	-0.078	0.725	0.012	0.914	0.925 (0.223-3.832)
Baseline MLR	-0.283	0.990	0.082	0.775	0.754 (0.108-5.244)
Baseline SII	-0.263	0.979	0.072	0.788	0.769 (0.113-5.239)
Baseline SES-CD	0.193	0.361	0.287	0.592	1.213 (0.598-2.460)

UST, Ustekinumab; BMI, body mass index; ALB, albumin; PLT, platelet; Hb, hemoglobin; CDAI, Crohn’s Disease Activity Index; CRP, C-reactive protein; ESR, erythrocyte sedimentation rate; PLR, platelet–lymphocyte ratio; MLR, monocyte–lymphocyte ratio; NLR, neutrophil–lymphocyte ratio; SII, systemic immune-inflammation index; SES-CD, Simple Endoscopic Score for Crohn’s Disease. Bold values indicate statistical significance (p < 0.05).

The glucocorticoid-free response rate at week 48 was 74.5% (117/157). Clinical remission rates at weeks 16 and 24 were 49.0% (77/157) and 62.4% (98/157), respectively. No serious adverse events were observed.

## Discussion

Since the advent of biologic agents, the number of treatment options for CD has substantially increased. Among these options, UST targets the IL-12/23 pathway to alleviate intestinal inflammation (6, 16). However, the identification of patients who are most suitable for UST treatment remains unclear. Given that multiple biologic agents are recommended in parallel as first-line therapies, the conventional trial-and-error approach delays disease control while increasing the economic and psychological burden on patients. In the present study, a systematic retrospective analysis of multicenter real-world data identified two readily accessible objective indicators—baseline neutrophil count (≤4.135×10^9^/L) and baseline PLR (<237.912)—as reliable predictors of “dominant responders” to UST.YV

The clinical remission rate at week 48 was significantly higher in patients with baseline neutrophil count ≤4.135×10^9^/L than in those with neutrophil count >4.135×10^9^/L (66.7% [80/120] vs. 33.3% [40/120]; *p* < 0.001). Similarly, patients with baseline PLR <237.912 achieved a significantly higher rate of clinical remission than those with PLR ≥237.912 (75% [90/120] vs. 25% [30/120]; *p* < 0.001).

Further subgroup analysis revealed that baseline neutrophil count ≤4.135×10^9^/L was significantly predictive of UST response in patients with prior anti-TNF-α treatment failure but not in those who received UST as initial therapy. This finding might reflect a specific association between the Th17/neutrophil axis in refractory CD and the response to UST. Patients with elevated neutrophil counts may have inflammation driven by the Th17/neutrophil axis. Neutrophils mainly mediate tissue damage by releasing neutrophil extracellular traps (NETs), reactive oxygen species (ROS) and proteolytic enzymes in CD intestinal inflammation (17). Neutrophils mainly mediate tissue damage in CD intestinal inflammation by releasing NETs, ROS, and proteolytic enzymes. In patients who failed anti-TNF-α treatment, their inflammatory mechanisms may have shifted or relied on the Th17/neutrophil axis. Patients with elevated neutrophil counts may have NETs-mediated tissue damage and intestinal epithelial barrier disruption, resulting in a poorer response to UST. By contrast, PLR <237.912 was significantly predictive of UST response in both subgroups, suggesting an association between global inflammatory burden and UST response. PLR incorporates platelet-related information and may reflect a broader state of immune activation, including thromboinflammatory processes, aligning with anti-inflammatory effects of UST beyond specific immune pathways.

The above evidence supports baseline PLR <237.912 and baseline neutrophil count ≤4.135×10^9^/L as specific predictors of UST response. The present findings also provide a stratified prediction strategy for clinical practice: among patients receiving UST as initial therapy, baseline PLR may be of primary importance, whereas among patients with anti-TNF-α treatment failure, baseline PLR and baseline neutrophil count should both be considered.

Our findings are consistent with increasing evidence that the systemic inflammatory state before treatment greatly influences the efficacy of biologic therapies (18, 19). A recent meta-analysis reported that peripheral blood NLR and PLR levels were significantly higher in patients with CD than in healthy individuals. Moreover, NLR and PLR both significantly differed between active and remission stages of CD; elevated levels were associated with greater disease activity and severity. Based on diagnostic accuracy analyses, NLR and PLR may serve as effective biomarkers for evaluating CD activity (20). Another study showed that higher preoperative NLR and PLR values in patients with CD were associated with major postoperative complications, increased risk of reoperation, and higher Clavien–Dindo scores. These findings suggest that blood-based inflammatory markers can facilitate patient selection and risk stratification when considering surgical intervention (21).

Neutrophils, lymphocytes, and platelets play important roles in the pathogenesis of mucosal injury in CD. Neutrophils are key mediators of intestinal inflammation; they contribute to host defense through phagocytosis, release of antimicrobial peptides, and formation of NETs (17). A principal mechanism by which neutrophils promote inflammation is the formation of NETs (22, 23). NETs consist of decondensed chromatin and granule proteins, including myeloperoxidase and neutrophil elastase, which can induce the secretion of proinflammatory cytokines such as TNF-α and IL-18, contributing to autoimmune processes (24). Experimental evidence suggests that inhibition of NET formation or function can alleviate chronic inflammation, reduce tissue damage, and improve symptoms associated with CD (25).

Autophagy and ROS have demonstrated involvement in NET formation (26), and ROS generation is essential for this process (27). Activated neutrophils release large amounts of ROS, causing oxidative damage to intestinal epithelial cells (28). Proteolytic enzymes released by neutrophils (e.g., myeloperoxidase and neutrophil elastase) can directly degrade the extracellular matrix and disrupt the integrity of the intestinal epithelial barrier. Therefore, an elevated baseline neutrophil count not only reflects inflammatory burden but may also indicate the potential for NET-mediated tissue damage. In the present study, low to moderate neutrophil counts (ountsphil^9^/L) predicted a favorable response to UST treatment. This association may be partly explained by lower NET formation capacity and reduced ROS burden at lower neutrophil levels, which facilitate intestinal repair after IL-12/23 blockade.

Th cell subsets and their cytokines play crucial roles in the pathogenesis of inflammatory bowel disease. An imbalance between effector T cells and regulatory T cells can trigger intestinal inflammation, given that effector subsets such as Th1, Th17, and Th9 are increased in patients with inflammatory bowel disease (29). Levels of IL-1b, IL-6 and TNF-α are increased in patients with CD (30). TNF-α can enhance the immune response of Th17 cells and promote the secretion of interferon (IFN)-γ, while IL-6 helps the differentiation of Th17 cells, thereby further regulating the destructive inflammatory response (31). In particular, Th1 cells (producing IFN-γ) and Th17 cells (producing IL-17) play central pathogenic roles in CD (32). Recent studies have identified a “Th1/17” cell subset that co-expresses T-box expressed in T cells (T-bet) and retinoic acid receptor–related orphan receptor C gene–encoded gamma t (RORC/γt) while producing both IL-17 and IFN-γ; this subset is greatly enriched in the intestinal mucosa of patients with CD and shows a close association with disease activity. Moreover, C-C chemokine receptor type 5 (CCR5)^+^ Th17 cells (pTh17 cells) preferentially accumulate at inflammatory sites in CD and can be induced by IL-23, suggesting that they are direct targets of IL-23 blockade (e.g., UST) (32). Thus, relative lymphocytosis associated with low PLR may reflect a state characterized by a lower proportion of pathogenic T cells or relatively preserved regulatory T cell function, allowing UST to more effectively restore immune balance.

Platelets are crucial for blood clotting. During the inflammatory process, they attach to endothelial cells to facilitate the recruitment of inflammatory cells, regulate cell adhesion and exudation, and activate neutrophils, etc. Additionally, platelets synthesize and release a large amount of pro-inflammatory cytokines and chemokines (33). During acute inflammation, IL-6 stimulates platelet differentiation, which not only exacerbates inflammation but also leads to thrombosis (34). Platelets contain IL-8, which can induce neutrophils aggregation and release superoxide, a process mediated by adhesion molecules, resulting in the aggregation of platelets and neutrophils (34). A study (35) showed that platelets activate neutrophils by releasing platelet microparticles containing high mobility group box 1, thereby promoting the formation of NET. CD is associated with a procoagulant state, which increases the risk of thromboembolic complications. The interaction between inflammation and coagulation enhances this risk by upregulating coagulation factors, downregulating natural anticoagulants, and impairing fibrinolysis. Patients with CD exhibit elevated levels of prothrombotic markers. Those with active disease often display reactive thrombocytosis, and platelet count is positively correlated with inflammatory markers such as CRP and IL-6 (36). Under inflammatory conditions, platelets become activated and express P-selectin and glycoprotein IIb/IIIa on their surface, forming platelet–leukocyte aggregates that promote leukocyte recruitment to inflamed tissues. Additionally, platelet-derived extracellular vesicles are enriched in phosphatidylserine and tissue factor, which enhance thrombin generation and contribute to a self-perpetuating cycle of inflammation and thrombosis (36). Elevated platelet count has been identified as an independent risk factor for venous thromboembolism in patients with inflammatory bowel disease; the risk of venous thromboembolism is two- to threefold higher in patients with CD than in the general population (37). Microthrombi form in the intestines and release inflammatory mediators. The elevated platelet count and activation of platelets can trigger a series of inflammatory responses by increasing vascular permeability and promoting the migration of white blood cells, which may exacerbate intestinal mucosal ischemia and potentially lead to irreversible intestinal damage (38). From this perspective, the relatively low platelet count reflected by a low PLR may indicate a lower prothrombotic and proinflammatory state, which contributes to a more favorable response to UST treatment.

PLR integrates platelet-related thromboinflammatory activity and lymphocyte-mediated adaptive immune regulation. A low PLR (<237.912) reflects a combined state of reduced platelet-driven proinflammatory and prothrombotic burden (36) and weaker enrichment of pathogenic T-cell subsets at sites of intestinal inflammation, or relatively preserved regulatory T-cell proportions (29). A reduced lymphocyte count may reflect two processes. First, lymphocytes (e.g., CD4^+^ T cells) may preferentially home to intestinal inflammatory sites, leading to a relative decrease in circulating lymphocyte levels; this decrease reflects redistribution, rather than absolute depletion (29). Second, enrichment of pathogenic T-cell subsets (Th1, Th17, Th1/17) may occur within inflamed tissue. This immunologic context may enhance UST efficacy by facilitating inhibition of Th1 and Th17 differentiation and function via IL-12/23 pathway blockade, contributing to higher clinical remission rates.

UST targets the common p40 subunit shared by IL-12 and IL-23, thus simultaneously inhibiting the Th1 and Th17 pathways (39). This dual mechanism of action is central to its therapeutic efficacy.

IL-17 is a potent mediator of neutrophil recruitment and activation (40). In patients with CD who have substantially elevated baseline neutrophil counts, disease activity may be driven by a highly active IL-23/Th17/neutrophil axis. Although UST acts by blocking the upstream IL-23 pathway, its efficacy may be limited when this axis is strongly activated and potentially accompanied by other inflammatory pathways. Additionally, Th17-mediated responses and subsequent neutrophil infiltration can disrupt the intestinal barrier and promote the release of proinflammatory mediators, creating a persistent inflammatory environment. Such an environment may further deplete lymphocytes and stimulate platelet production, contributing to elevated PLR. Conversely, patients with lower baseline neutrophil counts or lower baseline PLR may have a disease state that is more responsive to IL-12/23 blockade, facilitating better control of intestinal inflammation and higher rates of clinical remission.

Importantly, monoclonal antibodies targeting IL-17A have not demonstrated therapeutic benefit in clinical trials of CD; in some cases, they have been associated with disease exacerbation (41, 42). Although IL-17 inhibitors are effective in psoriasis and psoriatic arthritis, they may increase the risk of inflammatory bowel disease (43). This apparent contradiction highlights the complexity of immune regulation in CD. IL-17 appears to have both pathogenic and protective roles in intestinal inflammation. For instance, on one hand, IL-17 produced by Th17 cells promotes neutrophil recruitment and inflammation; on the other hand, it also contributes to the maintenance of intestinal epithelial barrier integrity and stimulates the production of antimicrobial peptides. Thus, complete blockade of IL-17A may impair mucosal barrier repair mechanisms and lead to adverse outcomes.

By contrast, UST targets the upstream IL-23 pathway. Its advantage arises from the fact that IL-23 is a key cytokine maintaining the pathogenic function of Th17 cells, although it is not the sole factor driving their differentiation. IL-23 blockade can selectively inhibit the inflammatory activity of pathogenic Th17 cells without completely abolishing the protective effects of IL-17. Moreover, IL-23 can induce the differentiation of pTh17 cells, a pathogenic T-cell subset characterized by high expression of IL-17 and IFN-γ, which is enriched in the intestinal mucosa of patients with CD and closely associated with disease activity (32).

Additionally, IL-12 blockade by UST inhibits Th1 differentiation and IFN-γ production (44). The established pathogenic model of CD emphasizes Th1-mediated inflammation, and IFN-γ has been shown to increase intestinal epithelial permeability. Therefore, the dual mechanism of UST—simultaneous inhibition of Th1 responses (via IL-12 blockade) and pathogenic Th17 responses (via IL-23 blockade)—allows immune network modulation in CD across two pathways. Such modulation may explain why UST remains effective in patients with prior anti-TNF-α treatment failure.

This study has several limitations. First, although it was conducted across multiple centers, the sample size was relatively small, and the retrospective design may limit the generalizability of the findings. Given its exploratory cohort design, the results require external validation in larger, prospective independent cohorts. Second, the proposed mechanistic explanations remain speculative and require further experimental studies to directly verify the roles of neutrophil and lymphocyte subsets in the response to UST treatment.

In conclusion, this study demonstrated that simple pretreatment blood parameters—low neutrophil count (ountophil^9^/L) and low PLR (<237.912)—can effectively identify “dominant responders” with a high likelihood of benefiting from UST, thus enhancing confidence in treatment selection among clinicians and patients. These findings represent a practical step toward individualized precision medicine. Baseline neutrophil count and baseline PLR are effective predictive biomarkers for clinical remission in patients with CD receiving UST. These low-cost and readily accessible indicators jointly reflect baseline inflammatory and immune statuses; they may facilitate the identification of patients most likely to benefit from UST, thereby optimizing clinical decision-making and improving patient outcomes.

## Data Availability

The original contributions presented in the study are included in the article/supplementary material. Further inquiries can be directed to the corresponding authors.
